# What is the frequency of anatomical variations and pathological findings in maxillary sinuses among patients subjected to maxillofacial cone beam computed tomography? A systematic review 

**DOI:** 10.4317/medoral.21456

**Published:** 2017-06-04

**Authors:** Javier Ata-Ali, Jose-Vicente Diago-Vilalta, María Melo, Leticia Bagán, Maria-Costanza Soldini, Chiara Di-Nardo, Fadi Ata-Ali, José-Félix Mañes-Ferrer

**Affiliations:** 1 Public Dental Health Service. Arnau de Vilanova Hospital, Valencia, Spain; 2 Department of Dentistry, European University of Valencia, Valencia, Spain

## Abstract

**Background:**

When considering dental implant rehabilitation in atrophic posterior sectors, the maxillary sinuses must be evaluated in detail. Knowledge of the anatomical variations and of the potential lesions found in these structures conditions the outcome of sinus lift procedures and therefore of the dental implants. A systematic review is made to determine the frequency of anatomical variations and pathological findings in maxillary sinuses among patients subjected to cone beam computed tomography (CBCT).

**Material and Methods:**

A PubMed (MEDLINE) literature search was made of articles published up until 20 December 2015. The systematic review was conducted based on the Preferred Reporting Items for Systematic Reviews and Meta-analysis (PRISMA). The quality of the studies included in the review was assessed using the Methodological Index for Nonrandomized Studies (MINORS).

**Results:**

The combinations of search terms resulted in a list of 3482 titles. Twenty-three studies finally met the inclusion criteria and were entered in the systematic review, comprising a total of 11,971 patients. The most common anatomical variations were pneumatization and sinus septa. The prevalence of maxillary sinus disease ranged from 7.5% to 66%. The most common pathological findings of the maxillary sinus were mucosal thickening, sinusitis and sinus opacification.

**Conclusions:**

Although the main indication of CBCT of the maxillary sinus in dentistry is sinus floor elevation/treatment planning and evaluation prior to dental implant placement, this imaging modality is increasingly also used for endodontic and periodontal purposes. There is no consensus regarding the cutoff point beyond which mucosal thickening of the maxillary sinus should be regarded as pathological, and the definition of maxillary sinusitis moreover varies greatly in the scientific literature. In this regard, international consensus is required in relation to these concepts, with a clear distinction between healthy and diseased maxillary sinuses.

** Key words:**Maxillary sinus, cone beam computed tomography, dental implant, maxillary sinus floor augmentation, sinus membrane, sinus floor elevation.

## Introduction

Implant placement in the posterior maxilla may be a challenging surgical procedure because of the reduced vertical bone height resulting from expansion of the maxillary sinus. Sinus floor elevation procedures are often needed to treat such bone deficiencies, in order to allow correct placement of dental implants (1). Apart from differences in indications, transcrestal and lateral window sinus augmentation procedures are predictable, and implants placed in grafted sinuses have high survival rates (2-4). Nevertheless, complications still occur, associated mainly with membrane perforation that is often caused by inadequate surgical planning or maneuvers (5). In this regard, perforation or damage of the Schneiderian membrane reportedly occurs in an average of 19.5% of the cases (up to 58.3%) (2).

The anatomical variability that may be found in the maxillary sinus has a strong impact upon the risk of sinus membrane perforation and subsequent implant failure. Computed tomography is considered the gold standard for sinus diagnosis, because of its ability to provide multiple sections through the sinus at different planes and allow visualization of bone and soft tissues (6). Barone *et al.* noted that membrane perforation might lead to graft migration and sinus infection. Thus, an intact Schneiderian membrane is desirable to ensure better vascularization, graft stability and environmental conditions for maturation of the inserted bone graft materials (7,8).

When considering dental implant rehabilitation in atrophic posterior sectors, the maxillary sinuses must be evaluated in detail. Knowledge of the anatomical variations and of the potential lesions found in these structures conditions the outcome of sinus lift procedures and therefore of the dental implants. Since the maxillary sinus is an anatomical structure that can be visualized by maxillary CBCT, the professionals performing such explorations must not only record the radiological findings for which CBCT is requested (dental implants, endodontics, periodontics, etc.) but should also evaluate the rest of the structures seen during the exploration. In this regard, the aim of the present systematic review was to answer the question: What is the frequency of anatomical variations and pathological findings in maxillary sinuses among patients subjected to maxillofacial cone beam computed tomography (CBCT)?

## Material and Methods

The Preferred Reporting Items for Systematic Reviews and Meta-analysis (PRISMA) statement was used in this study (9).

- PICO question

What is the frequency of anatomical variations and pathological findings in maxillary sinuses among patients subjected to maxillofacial cone beam computed tomography (CBCT)?

- Search Strategy for the Identification of Studies

The PubMed (MEDLINE) database of the United States National Library of Medicine was used for a literature search of articles published up until December 2015. The following search terms were used in different combinations: “cone beam computed tomography”, “mucosal thickness”, “sinus membrane” “maxillary sinus”, “CBCT”, “posterior maxilla”. Two examiners read the titles and abstracts of all studies, and no blinding was carried out regarding author names, journals or publication date. The search was completed with a review of the references of the selected articles in order to identify additional studies not found in the initial literature search.

In addition, a manual search (likewise up until December 2015) was made of the following journals: Clinical Implant Dentistry and Related Research, Clinical Oral Investigations, Clinical Oral Implants Research, Implant Dentistry, The International Journal of Oral and Maxillofacial Implants, Journal of Clinical Periodontology, Journal of Oral Implantology, Journal of Periodontology and Medicina Oral, Patología Oral y Cirugía Bucal.

-Study Selection Criteria

Before starting the study, a series of inclusion and exclusion criteria were established. Chosen full-text articles were assessed for the following inclusion criteria: randomized clinical trials, prospective cohort studies, controlled clinical trials and retrospective studies, with a sample size of ≥ 200 patients.

We excluded studies involving patients with congenital diseases (e.g., harelip and cleft palate) or maxillofacial traumatisms that could affect the region of the maxillary sinus. *In vitro* studies, animal studies, systematic reviews and case reports were also excluded. Authors were contacted for clarification of missing information when necessary. No restrictions were placed on the year or language of publication. All articles selected from the electronic and manual searches were independently assessed by the first and second authors of the present study, according to the established inclusion criteria. Any disagreements between the reviewing authors were resolved by consensus, or by consulting the last signing author of the study. The level of agreement between the two reviewing authors was assessed using the Cohen kappa statistic.

- Data Extraction and Assessment of Methodological Quality

Data were independently extracted from the included studies by two reviewers (JAA and JVDV). A third reviewer (JFMF) was consulted in the event of any disagreement.

Two authors independently evaluated the quality of the studies included in the systematic review using the Methodological Index for Nonrandomized Studies (MINORS) (10). The MINORS scale includes the following points: (a) a clearly stated aim; (b) inclusion of consecutive patients; (c) prospective collection of data; (d) appropriate endpoints; (e) unbiased assessment; (f) a follow-up period; (g) losses to follow-up of < 5%; and (h) prospective calculation of the study size. The items on the MINORS scale are scored as 0 (not reported), 1 (reported but inadequate) or 2 (reported and adequate). We defined study quality as poor (< 5), fair (6-10) or good (> 11). The level of agreement between the two reviewing authors was assessed using the Cohen kappa statistic.

## Results

- Study selection

**Figure 1:**
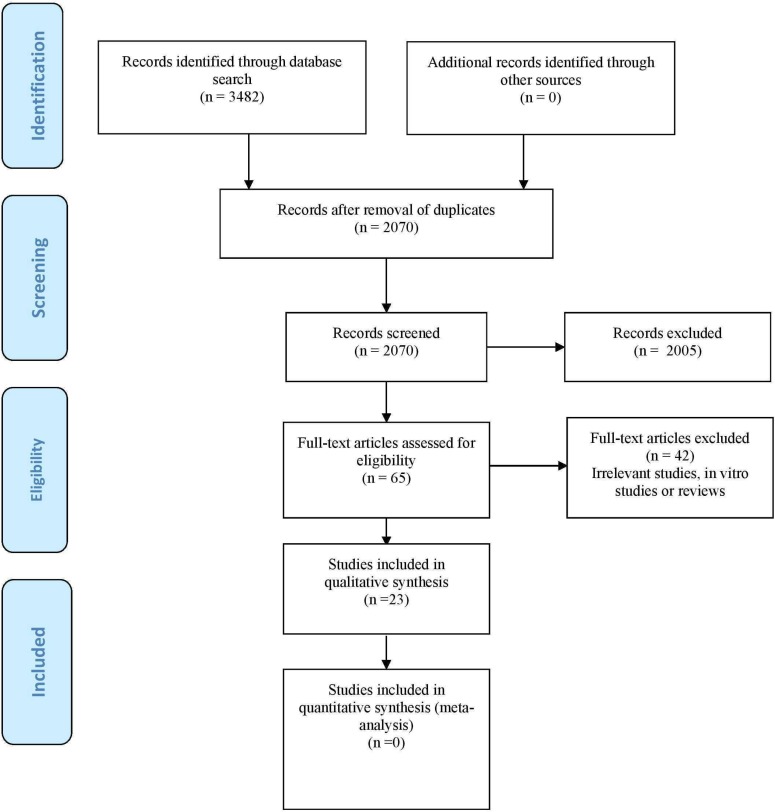
Prisma® flow chart of the search processes and results.

The combinations of search terms resulted in a list of 3482 titles. Of these, 1412 were found to be duplicated; as a result, 2070 references were reviewed. Subsequently, 2005 papers were excluded on the basis of the evaluation of the title and abstract, thus leaving 65 articles for eligibility assessment. Twenty-three publications finally met the inclusion criteria and were thus selected for inclusion in the systematic review (Fig. 1). The main indication of maxillary sinus CBCT was sinus floor elevation/treatment planning and evaluation prior to dental implant placement (50%), followed by exploration for endodontic and periodontal purposes. In only two articles was CBCT indicated for orthodontic evaluation. Inter-rater reliability based on the kappa statistic was 0.89.

- Assessment of study quality

Two reviewers independently and in duplicate evaluated the quality of the included studies ( Table 1) as part of the data extraction process. Any disagreements were resolved by consensus or by consulting the last signing author of the present study. The mean score for the global studies was 10 (range 7-12). Of the 23 studies finally included, 11 (11,13,15,17,21-26,28) were of fair quality, with a score of 6-10 points, and 12 studies (1,12,14,16,18-20,27,29-32) were of good quality, with a score of ≥ 11 points. Agreement between the reviewers for risk of bias assessment as evidenced by the kappa statistic was 0.90.

**Table 1:**
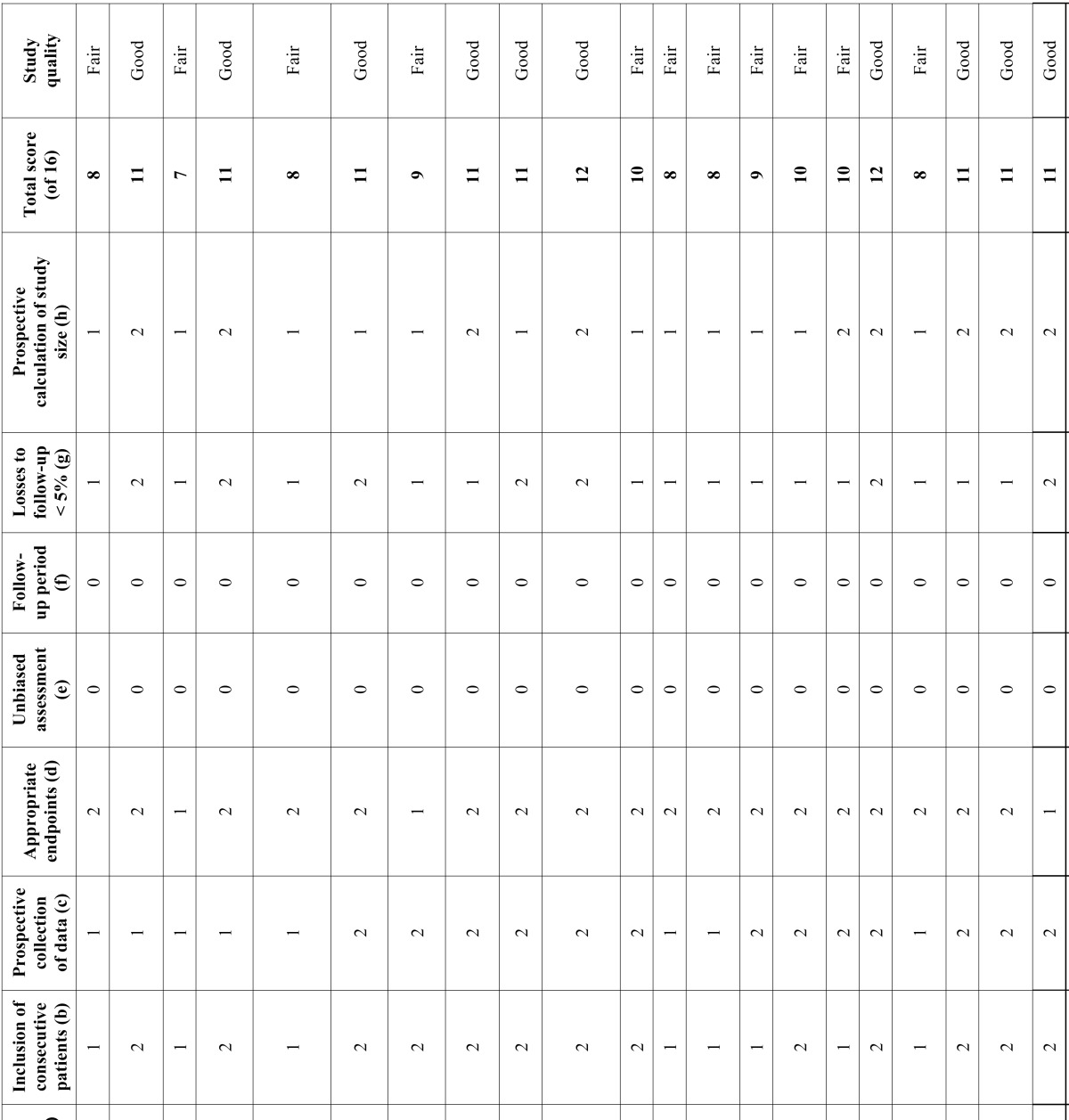
Quality assessment scores using the Methodological Index for Nonrandomized Studies (MINORS).

- Description of the studies 

One cross-sectional study and 22 retrospective studies were included in the systematic review. The demographic data (patient age and sex) and information referred to the maxillary sinus findings of the publications are summarized in  Table 2,  Table 2 continue. In the present systematic review we included a total of 11,971 patients.

**Table 2:**
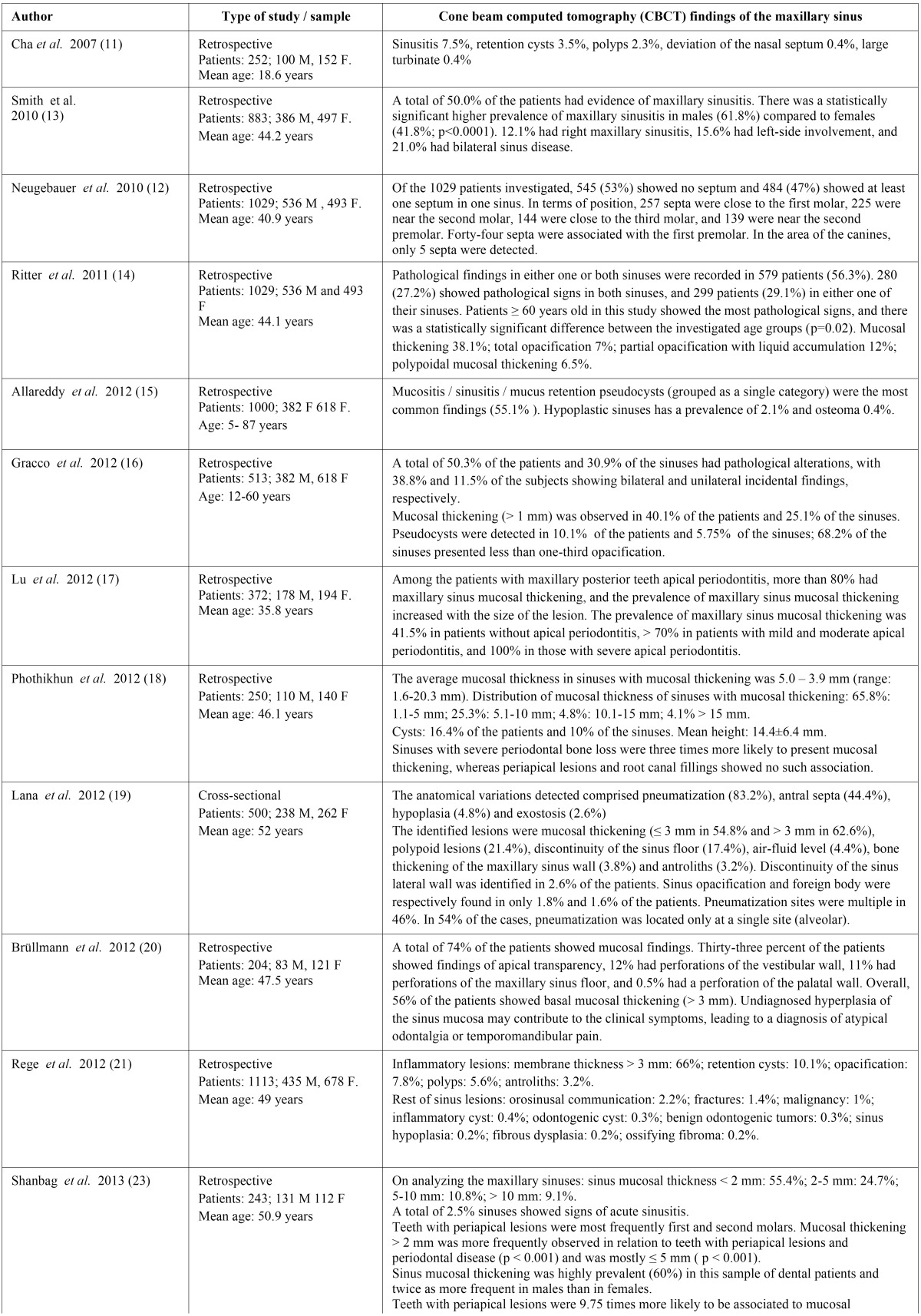
Demographic data and information referred to the findings of the maxillary sinus.

**Table 2 continue:**
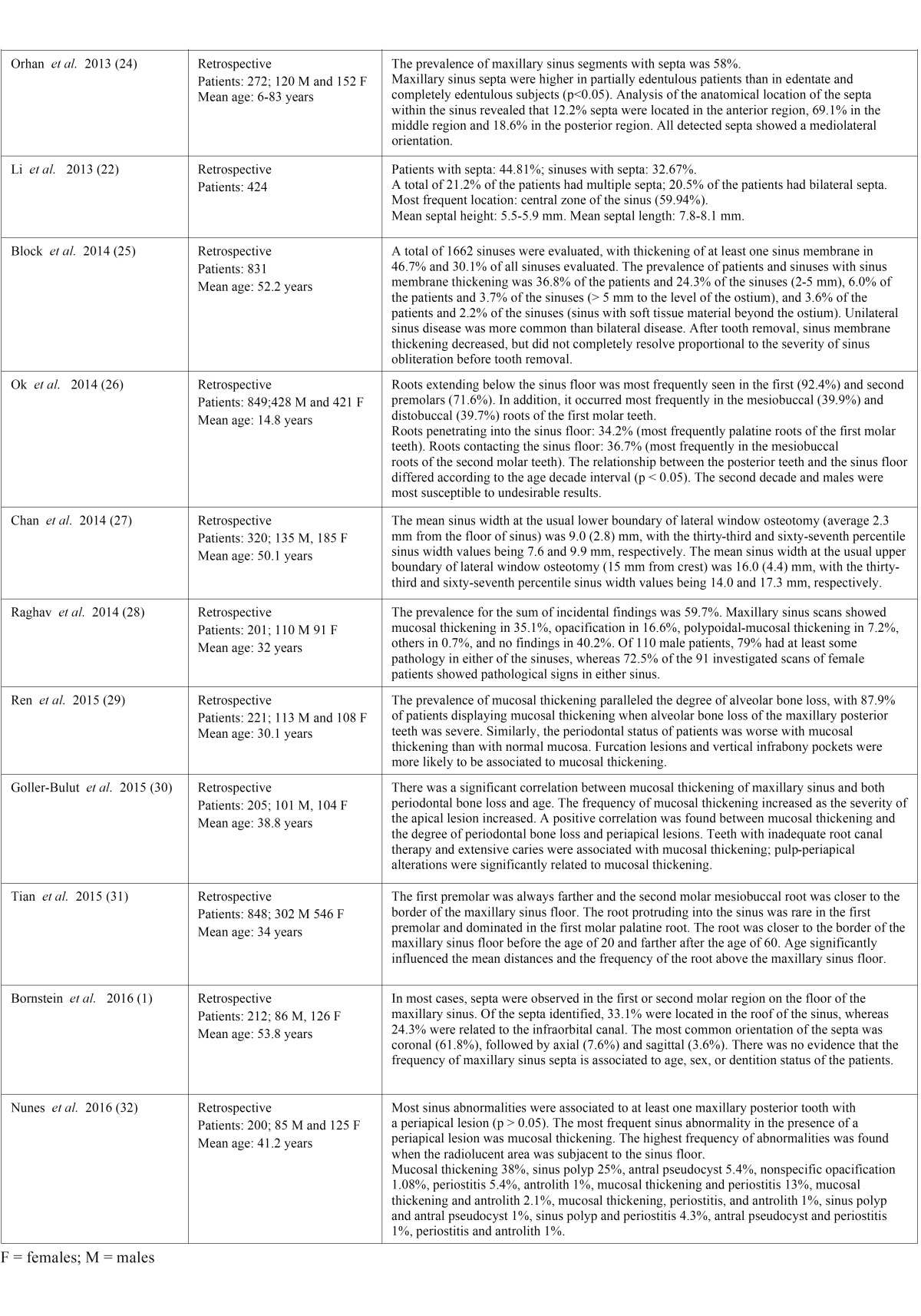
Demographic data and information referred to the findings of the maxillary sinus.

- Anatomical variations of the healthy maxillary sinus 

Over 50% of the included studies (n=15; 65.2%) identified anatomical variations of the healthy sinus.

- Sinus septa

Five studies (1,12,9,22,24) offered information on the prevalence of maxillary sinus septa, which ranged from 33.2-58%. Most patients (12) with septa showed one septum in one sinus (24.6%); 13.7% showed one septum in each sinus. Other combinations (up to three septa per sinus) were found in 8.7% of the patients. The septa were commonly found in the region of the first and second molars (60.7%) (1).

- Relationship between the roots of maxillary teeth and the maxillary sinus floor

Several studies (26,30,31) offered data on the relationship between the roots of maxillary tooth and the maxillary sinus floor. In one publication (26) involving 5166 maxillary premolars and molars, the roots extended below the sinus floor mainly in relation to the first (92.4%) and second premolars (71.6%); the roots penetrated into the sinus floor in 34.2% of the cases, and contacted the sinus floor in 36.7% of the cases.

- Other anatomical variations

In one study (19) involving 500 patients, the most frequent anatomical variation detected was pneumatization (83.2%). This same study (19) described the presence of exostosis in 2.6% of the cases, with unilateral location in 84.6% of the cases. Three publications (15,19,21) offered information on the prevalence of maxillary sinus hypoplasia, which ranged from 0.2-4.8%.

- Maxillary sinus disease

• Thickening of the sinus membrane 

The prevalence of mucosal thickening ranged between 35.1-66% (13,20,21,25,28,32). The cutoff point beyond which thickening is considered pathological is normally 1-3 mm. However, the prevalence of maxillary sinus mucosal thickening was 41.5% in patients without apical periodontitis, over 70% in patients with mild and moderate apical periodontitis, and 100% in those with severe apical periodontitis (17).

• Sinusitis and mucosal cysts

The prevalence of maxillary sinusitis ranged from 7.5-50% (11,13,20). The prevalence of mucosal cysts in turn ranged from 3.5-16.4% (11,16,18,32).

• Opacification of the maxillary sinus

Partial or complete opacification showed a prevalence of 1.8-68.2% (14,16,19,21,28,32). In one study (16) involving 1026 maxillary sinuses, 68.2% presented opacification of less than one-third of the sinus.

- Antroliths and polyps

The prevalence of antroliths ranged from 1% (32) to 3.2% (19,21), while the frequency of polyps ranged from 2.3% (11) to 25% (32).

- Other lesions of the maxillary sinus 

One publication (21) on the frequency distribution of sinus abnormalities in images from 703 patients and 1406 sinuses documented malignant tumors in 1% of the cases, benign odontogenic tumors in 0.3%, fibrous dysplasia in 0.2%, and ossifying fibroma in 0.2%. Another study (19) reported a foreign body prevalence of 1.6%.

## Discussion

The present systematic review has examined the scientific evidence with a view to determining the frequency of anatomical variations and pathological findings of the maxillary sinuses in patients subjected to maxillofacial CBCT. Twenty-three publications were included in our systematic review, comprising a total of 11,971 patients. The most common anatomical variations were pneumatization and sinus septa. The prevalence of sinus maxillary disease ranged from 7.5-66% - the most common disorders being mucosal thickening, sinusitis and opacification.

Maxillary sinus septa are barriers of cortical bone that divide the maxillary sinus floor into multiple compartments known as recesses (33). Septa have become increasingly important after the introduction of sinus floor augmentation surgery, since their presence may complicate both creation and inversion of the access window in the lateral sinus wall, as well as elevation of the sinus membrane from the bony sinus floor (34). Septa with a low height (up to 2 mm) do not require further treatment (12), because in routine cases the membrane can be elevated without further procedures. The shapes of the instruments determine the need to remove parts of the septa to release the sinus mucosa from the bone. Medium-size septa require resection, because the palatal area of the sinus cavity cannot be reached by the instruments. High septa in turn lead to partial or complete separation of the sinus cavity, requiring the preparation of two or even three cavities. The orientation of septa limits the mobility of the sinus instruments, resulting in a need for increased vestibular access for complication-free handling without creating uncontrolled pressure on the membrane (35). In our systematic review, the prevalence of maxillary sinus septa ranged from 33.2-58%.

There is no consensus regarding the cutoff point beyond which mucosal thickening of the maxillary sinus is considered pathological. In this regard, different authors define pathological thickening as ≥ 1 mm (16), ≥ 2 mm (17) or > 3 mm (19,21). In a study involving 831 patients, the prevalence of sinus membrane thickening was 36.8% (2-5 mm) of the patients and 24.3% of the sinuses; 6.0% of the patients and 3.7% of the sinuses presented more than 5 mm to the level of the ostium, and 3.6% of the patients and 2.2% of the sinuses presented soft tissue material beyond the ostium (25). A study (18) of CBCT images of 500 maxillary sinuses found the average mucosal thickness in sinuses with mucosal thickening to be 5.0-3.9 mm (range 1.6-20.3 mm). The majority of sinuses with mucosal thickening had a mucosal thickness of < 5 mm. Severe periodontal bone loss was significantly associated to mucosal thickening of the maxillary sinus. Sinuses with severe periodontal bone loss were three times more likely to have mucosal thickening (18), while Brüllman *et al.* recorded an odds ratio (OR) of 10.2 for the association of periodontitis to visibly thickened mucosa (20).

The most common causes of odontogenic sinusitis are dental abscesses and periodontal disease perforating the Schneiderian membrane. It is estimated that 10-12% of all cases of maxillary sinusitis have a dental origin (18). Sinusitis is the leading cause of mucosal thickening in symptomatic individuals (18). The relationship between dental infections and maxillary sinusitis is well established (36). The cause of mucosal thickening among asymptomatic individuals, however, remains unclear. In a study (37) of 190 patients treated for unilateral paranasal sinusitis, odontogenic infection was implicated in approximately 70% of the cases of unilateral paranasal sinusitis. Odontogenic maxillary sinusitis can be difficult to diagnose, and imaging exploration under various conditions is recommended. The definition of maxillary sinusitis varies greatly in the scientific literature. This is reflected by the findings of our systematic review, where the prevalence of maxillary sinusitis ranged widely from 7.5-50% (11,13,20). According to some authors such as Smith *et al.* (13), sinusitis is defined as any evident thickening of the mucosa in the maxillary sinus, with a prevalence of 50.0% in a series of 883 patients. Iatrogenic perforation of the maxillary sinus membrane during membrane elevation increases the chance of postoperative sinusitis, owing to bacterial graft contamination or graft migration into the sinus cavity (38). With appropriate treatment, intraoperative sinus membrane perforations did not represent an elevated risk for implant loss, infectious complications or displacement of graft material (39). In a study comprising 407 sinus grafts in 300 patients (39), the prevalence of Schneiderian membrane perforation was 8.6%, and was significantly correlated to the presence of sinus septa (OR = 4.8), smoking (OR = 4.8) and decreased residual bone height (OR = 0.01). The frequency of postoperative sinusitis was significantly greater for sinus membrane perforation (OR = 10.5) and in smokers (OR = 12.3).

Panoramic radiography has been used as a routine screening tool for evaluation of the maxillomandibular complex (40). However, panoramic radiography has limitations in diagnosing sinus disorders, and computed tomography remains the most effective diagnostic technique (41). Martínez-González *et al.* (41) compared panoramic radiography and computed tomography in evaluating 84 maxillary sinuses, and found panoramic radiography to have limitations in diagnosing changes in the maxillary sinus, whereas computed tomography seemed to be a better imaging tool. In a study published by Wolff *et al.* (42) in a total of 253 patients subjected to both panoramic radiographic and CBCT analysis, CBCT imaging provided significantly more surgically relevant information in cases of implant dentistry and maxillary sinus diagnosis. Visualization quality of the maxillary sinus and bony structures in CBCT appears to be similar to that afforded by computed tomography. However, CBCT generates high-resolution isotropic volume data and therefore could offer benefits in evaluating the bony aspects of the maxillary sinus thanks to the use of a lower radiation dose (14).

- Limitations

The results of our systematic review cannot be extrapolated to the general population, since the great majority of the patients in the included studies corresponded to CBCT explorations performed in the context of dental implant planning, i.e., the patients were typically elderly individuals with missing teeth in the upper maxilla. The main limitation of our systematic review is the fact that the results were not integrated in a quantitative analysis, thereby precluding the conduction of a meta-analysis. This was mainly due to significant heterogeneity between publications referred to disease definitions (with multiple definitions regarding mucosal thickening and sinusitis of the maxillary sinus), measured outcomes and other aspects. Another aspect that also must be taken into account on interpreting the results is the fact that 12 studies were of good quality, while 11 were of fair quality - the mean MINORS score being 10 out of 16 (range 7-12). We were not able to take the “comparator” component C of the PICO question into account. In some cases the PICO question cannot be applied in its entirety, particularly in the case of anatomical studies. Huang *et al.* (43) reported that in some cases it is difficult to encode certain question classes without modifying the existing PICO structure or introducing counterintuitive elements. The PICO representation is unable to capture anatomical relations that may be relevant in a clinical question. There is no slot in the PICO framework capable of capturing “body parts”.

## Conclusions

Although the main indication of maxillary sinus CBCT is sinus floor elevation/treatment planning and evaluation prior to dental implant placement, this imaging modality is increasingly also used for endodontic and periodontal purposes. There is no consensus regarding the cutoff point beyond which mucosal thickening of the maxillary sinus should be regarded as pathological, and the definition of maxillary sinusitis moreover varies greatly in the scientific literature. In this regard, international consensus is required in relation to these concepts, with a clear distinction between healthy and diseased maxillary sinuses in order to facilitate comparisons between studies.
